# SNOMED CT Concept Hierarchies for Computable Clinical Phenotypes From Electronic Health Record Data: Comparison of Intensional Versus Extensional Value Sets

**DOI:** 10.2196/11487

**Published:** 2019-01-16

**Authors:** Ling Chu, Vaishnavi Kannan, Mujeeb A Basit, Diane J Schaeflein, Adolfo R Ortuzar, Jimmie F Glorioso, Joel R Buchanan, Duwayne L Willett

**Affiliations:** 1 University of Texas Southwestern Medical Center Dallas, TX United States; 2 University of Wisconsin School of Medicine and Public Health Madison, WI United States

**Keywords:** SNOMED CT, value sets, clinical phenotypes, population health, pragmatic clinical study

## Abstract

**Background:**

Defining clinical phenotypes from electronic health record (EHR)–derived data proves crucial for clinical decision support, population health endeavors, and translational research. EHR diagnoses now commonly draw from a finely grained clinical terminology—either native SNOMED CT or a vendor-supplied terminology mapped to SNOMED CT concepts as the standard for EHR interoperability. Accordingly, electronic clinical quality measures (eCQMs) increasingly define clinical phenotypes with SNOMED CT value sets. The work of creating and maintaining list-based value sets proves daunting, as does insuring that their contents accurately represent the clinically intended condition.

**Objective:**

The goal of the research was to compare an intensional (concept hierarchy-based) versus extensional (list-based) value set approach to defining clinical phenotypes using SNOMED CT–encoded data from EHRs by evaluating value set conciseness, time to create, and completeness.

**Methods:**

Starting from published Centers for Medicare and Medicaid Services (CMS) high-priority eCQMs, we selected 10 clinical conditions referenced by those eCQMs. For each, the published SNOMED CT list-based (extensional) value set was downloaded from the Value Set Authority Center (VSAC). Ten corresponding SNOMED CT hierarchy-based intensional value sets for the same conditions were identified within our EHR. From each hierarchy-based intensional value set, an exactly equivalent full extensional value set was derived enumerating all included descendant SNOMED CT concepts. Comparisons were then made between (1) VSAC-downloaded list-based (extensional) value sets, (2) corresponding hierarchy-based intensional value sets for the same conditions, and (3) derived list-based (extensional) value sets exactly equivalent to the hierarchy-based intensional value sets. Value set conciseness was assessed by the number of SNOMED CT concepts needed for definition. Time to construct the value sets for local use was measured. Value set completeness was assessed by comparing contents of the downloaded extensional versus intensional value sets. Two measures of content completeness were made: for individual SNOMED CT concepts and for the mapped diagnosis clinical terms available for selection within the EHR by clinicians.

**Results:**

The 10 hierarchy-based intensional value sets proved far simpler and faster to construct than exactly equivalent derived extensional value set lists, requiring a median 3 versus 78 concepts to define and 5 versus 37 minutes to build. The hierarchy-based intensional value sets also proved more complete: in comparison, the 10 downloaded 2018 extensional value sets contained a median of just 35% of the intensional value sets’ SNOMED CT concepts and 65% of mapped EHR clinical terms.

**Conclusions:**

In the EHR era, defining conditions preferentially should employ SNOMED CT concept hierarchy-based (intensional) value sets rather than extensional lists. By doing so, clinical guideline and eCQM authors can more readily engage specialists in vetting condition subtypes to include and exclude, and streamline broad EHR implementation of condition-specific decision support promoting guideline adherence for patient benefit.

## Introduction

### Overview

Given widespread adoption of electronic health records (EHRs) certified to follow terminology standards, why does achieving interoperable clinical phenotype definitions remain challenging? Practical approaches to analytic interoperability among EHR-originated datasets would provide value both for population health analytics and clinical research [1-3]. Clinical registries define most patient subpopulations—important clinical phenotypes—by either a shared condition or a shared exposure (eg, to a type of procedure or medication) [4]. EHRs now encode patient conditions in clinical terminologies mapped to SNOMED CT, an international comprehensive clinical terminology [5-7]. By federal standard, exchanging patient conditions (problems) between EHRs via health information exchanges employs SNOMED CT concepts.

Accordingly, clinical quality measures derived from EHR data increasingly define clinical phenotypes with SNOMED CT concept value sets, analogous to the International Classification of Diseases (ICD) code value sets traditionally defined for claims data. Initial SNOMED CT value sets primarily have taken an “extensional” form—that is, an enumerated list of terms—in keeping with the long-standing structure of ICD code value sets [8]. But SNOMED CT, being a polyhierarchical ontology, affords the powerful option of employing rule-based or “intensional” value sets leveraging the relationships within the ontology. Such intensional value sets can more concisely identify included and excluded subtypes of a clinical condition by referring to SNOMED CT’s hierarchical “is a” supertype-subtype (parent-child) relationships. Those subtype relationships can be a close match to clinicians’ thinking about clinical phenotypes and the subtypes of conditions they wish to be included or excluded. In a report on 125 such hierarchy-based value sets, we’ve shown they also are simple to create in an EHR and employ in an analytic data warehouse [9].

In this study, we examined value sets defining 10 conditions referenced by 2018 Centers for Medicare and Medicaid Services (CMS) high-priority electronic clinical quality measures (eCQMs) for adults. We compare corresponding intensional versus extensional SNOMED CT value sets for their conciseness, time to construct, and completeness of SNOMED CT concept inclusion. We also compare their completeness in covering the SNOMED CT-mapped clinical terms selectable by clinicians within the EHR as patient Problem List entries and encounter diagnoses, since those selections ultimately drive clinical phenotypes for population health activities and pragmatic clinical studies employing EHR source data.

### Extensional SNOMED CT Value Sets

#### Why Value Sets?

Transactional source data from administrative and clinical information systems typically include diagnosis information encoded in either ICD or SNOMED CT. Value sets of specified ICD or SNOMED CT terms define conditions (clinical phenotypes) for use in clinical guidelines, clinical quality measures, and patient registries [4]. Two categories of conditions commonly need to be defined: (1) one or more primary, population-defining conditions and (2) comorbid conditions used for exclusions and/or risk stratification.

#### Why SNOMED CT Value Sets?

To be certified for the Meaningful Use program in the United States, EHRs must be able to transmit patient diagnosis information to another EHR using SNOMED CT–encoded concepts [10,11]. Thus, in most EHRs clinicians enter patient conditions onto their Problem List by using either SNOMED CT directly, or, more commonly, a clinician-friendly clinical terminology premapped to SNOMED CT concepts. Both methods enable preserving a higher level of clinical fidelity and relevant clinical detail than ICD does due to the enhanced clinical specificity of SNOMED CT [9].

#### Why Extensional SNOMED CT Value Sets?

Extensional value sets refer to simple lists of codes or concepts. ICD value sets traditionally have been constructed this way, in keeping with the structure of ICD [12,13]. As the need for SNOMED CT value sets arose, the same approach was continued. The innovative Value Set Authoring Tool made available in 2013 by the Value Set Authority Center (VSAC) initially supported only creation of extensional value sets [14].

### Challenges with Extensional SNOMED CT Value Sets

Extensional value sets, as specifically enumerated lists, are brittle and prone to “break” or become stale with updates to the underlying terminology. SNOMED CT updates can include addition of new clinical concepts or refining an existing concept by creating or expanding its “descendant” concepts. Preexisting extensional value set lists cannot handle these automatically and may require frequent reupdating after new SNOMED CT version releases, followed by reimportation or copying into every EHR or other system employing the value set.

Some extensional value sets include many items, which inhibits rapid human comprehension of exactly which subtypes of a given clinical condition are being included and excluded. Thus clinical vetting of such value sets becomes laborious. Similarly, construction of the value set and performing quality assurance are correspondingly difficult and labor-intensive. Inaccuracies in value sets can significantly affect clinical quality measure calculations [15].

### Intensional Value Sets of SNOMED CT Concept Hierarchies

SNOMED CT intensional value sets, by contrast, are rule-based and leverage the polyhierarchy structure of SNOMED CT. That is, one can include or exclude an entire “tree” of real-world condition subtypes via a single reference to a SNOMED CT concept and all its descendants. Combining such tree references with simple Boolean logic (or with SNOMED CT Expression Constraint Language) enables efficient definition of a desired clinical phenotype [9]. For instance, osteoporosis and all of its subtypes can be defined by reference to one SNOMED CT concept (SCT ID 64859006 Osteoporosis) and all its descendants. The corresponding extensional list would require 42 SNOMED CT concepts to fully define. In turn, in our EHR 2287 diagnosis clinical terms map to this single SNOMED CT concept hierarchy; a clinician selecting any one of these for a patient’s diagnosis would automatically include them in the broad computable clinical phenotype of osteoporosis.

Possible benefits of SNOMED CT intensional value sets include closely matching how clinicians think about what condition subtypes to include or exclude from a given clinical phenotype. Being able to reference the entire tree of a concept’s descendants enables far simpler, succinct value set definitions that are easier to understand and construct. Additionally, they should be more resilient to change and less likely to omit descendants and break with future SNOMED CT concept additions. Consequently, intensional value sets have potential to be simultaneously simpler and more complete and thus more useful for population health analytics and clinical research using EHR data.

### Objective of the Study

For each of 10 conditions (clinical phenotypes), evaluate the differences between an intensional (concept hierarchy-based) versus extensional (list-based) SNOMED CT value set approach in (1) conciseness, (2) time to create, and (3) completeness of both SNOMED CT concepts included and relevant clinical terms available for clinician selection in an EHR.

## Methods

### Selection of Value Sets

Value sets included in this study were identified starting from the CMS website for choosing Merit-Based Incentive Payment System (MIPS) quality measures [16]. MIPS measures were filtered for high-priority measures and data submission method of EHR, yielding 21 candidate measures. Four measures covering the following 4 common adult conditions were selected: hypertension, diabetes mellitus, depression, and prostate cancer.

Next, the online VSAC “search value sets” feature was employed to find condition-defining SNOMED CT value sets for these measures [17]. Value sets were first filtered for CMS eCQM Release = “eCQM Update 2018 EP-EC and EH” and Code System = “SNOMEDCT.” Then each of the eCQMs was selected individually, displaying the related SNOMED CT value sets. Any value sets specifying a condition (diagnosis) were included, yielding an initial total of 12 SNOMED CT extensional value sets (see Multimedia Appendix 1).

### Software

Creation of EHR vendor-neutral SNOMED CT intensional value sets and automatic derivation of extensional value sets were both done using Symedical (Clinical Architecture LLC), a clinical terminology management and mapping software tool for health care professionals. SNOMED CT intensional value sets (groupers) for EHR-based registry and clinical decision support functionality were created using the grouper management features of our EHR, Epic (Epic Systems Corporation). The clinical terminology vocabulary within University of Texas Southwestern Medical Center’s Epic EHR during this study was the proprietary IMO Problem IT terminology, version 2018 R1 (Intelligent Medical Objects Inc), mapped to the SNOMED CT International Edition July 2017 release and the SNOMED CT US Edition September 2017 release.

### Procedures

Using the VSAC website’s “export value set results” feature, the list of codes for each SNOMED CT extensional value set was exported to Excel (Microsoft Corp) for subsequent comparison.

Comparable intensional (rule-based) value set diagnosis groupers for these conditions were established in our EHR. The majority already existed, having been created for disease registries and/or clinical decision support [1]; two were newly created for this study (pain related to prostate cancer, personality disorder). Identically matching intensional value sets were then created in Symedical (in addition to Epic) and the time to create each intensional value set recorded.

To enable meaningful direct comparison with intensional value sets, two combinations of VSAC value sets were performed prior to comparing the SNOMED CT concept lists: (1) chronic kidney disease, stage 5, (CKD-5) was combined with end-stage renal disease (ESRD) since clinically they refer to the same condition, and so only one intensional value set covered both, and (2) major depression including remission was combined with dysthymia, as together they constitute the condition of depressive disorders covered by a single intensional value set. This yielded a final set of 10 clinical conditions for comparison. The eCQMs, VSAC value set identifiers, and extensional value set contents are available in Excel format in Multimedia Appendix 1.

The pregnancy value set (2.16.840.1.113883.3.526.3.378) downloaded from VSAC was found to include concepts focused on pregnancy itself but in general did not include concepts for complications or disorders of pregnancy. Our existing EHR-based intensional value set for pregnancy deliberately included the latter to provide a broad net for identifying any pregnant patients via EHR-entered diagnoses and problems. Accordingly, to better match the VSAC contents, we constructed a second narrow intensional value set for pregnancy based on the pregnancy conditions listed in the VSAC extensional value set by deliberately omitting SNOMED CT concepts for pregnancy-related conditions (eg, complication occurring during pregnancy, disorder of pregnancy). The VSAC extensional pregnancy value set was compared separately with both the broad and the narrow intensional pregnancy value sets.

For each intensional value set, a corresponding extensional value set list was automatically derived using Symedical (ie, a list of all included SNOMED CT concept descendants). These derived extensional value sets were downloaded and stored for subsequent analysis. The intensional value sets and corresponding derived extensional value sets are available in Excel format in Multimedia Appendix 2.

### Measures and Outcomes

#### Value Set Definition Conciseness

Conciseness of value set definition was measured simply by the number of SNOMED CT concepts needed to fully define the set, either as a list (extensional value set) or the number of concepts in the defining rule (intensional value set). A dimensionless ratio to define was calculated in two forms:

Ratio to define (download) = (# concepts in VSAC-downloaded extensional value set) / (# concepts in intensional value set defining rule)

Ratio to define (derived) = (# concepts in derived extensional value set) / (# concepts in intensional value set defining rule)

#### Time to Create

The time to create each of 11 intensional value sets (including both pregnancy value set versions) as well as 3 of the extensional value sets (CKD-5 & ESRD, prostate cancer, pain related to prostate cancer) in Symedical was measured. From this a best-fit linear equation was derived: time (min) = 0.4177*(# SNOMED CT concepts) + 3.8707. This corresponds to an obligate time of just under 4 minutes to create any value set (eg, for configuring basic common settings) plus approximately 0.42 minutes (25 seconds) to add each SNOMED CT concept. The time to create the remaining extensional value sets was estimated using this equation.

The difference in time to create an extensional versus an intensional value set was calculated as (time to create extensional value set) – (time to create intensional value set), expressed in minutes. The dimensionless ratio was calculated as (time to create extensional value set) / (time to create intensional value set).

#### Completeness: SNOMED CT Concepts

For each of 10 conditions, the list of SNOMED CT concepts included in the VSAC-downloaded set and the intensional-derived set were compared. The total number of concept discrepancies present in one set and not the other was assessed by summing two discrepancy types:

- Number of concepts present in the VSAC-downloaded set but not in the intensional-derived set
- Number of concepts present in the intensional-derived set but not in the VSAC-downloaded set

Since virtually all of the SNOMED CT concepts in the downloaded extensional value sets were included in the corresponding intensional-derived value set, the ratio of the two was calculated as: (# concepts in intensional-derived set) / (# concepts in VSAC-downloaded set), expressed as a number greater than 1. The percentage of SNOMED CT concepts included in the downloaded extensional value set was calculated as: (# concepts in VSAC-downloaded set) / (# concepts in intensional-derived set), expressed as a percentage.

#### Completeness: Electronic Health Record Clinical Term Coverage

To evaluate the impact of condition-specific discrepancies, value sets were created in the EHR in both an intensional form (existing) and an extensional form (to exactly match the VSAC list of concepts, without including descendants). The EHR automatically creates a compiled list of IMO-sourced clinical terms mapped to the SNOMED CT value set. These IMO clinical terms comprise the diagnoses visible to clinicians for selection as Problem List entries and as diagnoses to associate with patient orders, encounters, and professional charges. The number of clinical terms compiled for each intensional and extensional value set was recorded. Comparisons were then performed on the number of clinical terms available for selection by clinicians in the EHR that would result in patient inclusion in a given clinical phenotype.

Just as for SNOMED CT concept completeness, the ratio of the two was calculated as (# clinical terms from intensional-derived set) / (# clinical terms from VSAC-downloaded set), expressed as a number greater than or equal to 1. The percentage of clinical terms covered by the downloaded extensional value set was calculated as (# clinical terms from VSAC-downloaded set) / (# clinical terms from intensional-derived set), expressed as a percentage.

## Results

### Overall Format of Result Tables

Tabulated comparisons by each of the 10 conditions follow. Summary calculated measures are included at the bottom of each table. In addition to overall sums and ratios, the median of the 10 condition-specific values was selected as the primary measure of central tendency. This method was chosen a priori to avoid the potential for skew if one or more conditions exhibited marked difference from the others or contained many more concepts. The minimum, maximum, and range across the 10 conditions are also reported.

For pregnancy, both the narrow and broad definitions are shown in tabular form; however, only the more narrow intensional value set based on the CMS extensional value set was used in all summary calculations (to avoid double-counting). Use of the narrow pregnancy definition reduces the reported differences between intensional and extensional value sets so that the summary findings and conclusions shown are conservative. Were the broad pregnancy definition selected instead, the magnitude of effects would be larger. All tables are available in Excel format in Multimedia Appendix 3.

### Value Set Conciseness

We expected that intensional value sets should be more concise to construct by leveraging the hierarchical supertype-subtype structure of SNOMED CT. Table 1 shows that the median number of SNOMED CT concepts employed to define a condition with the VSAC value sets was 21.5 concepts versus only 3.0 for intensional value sets.

**Table 1:** Clinical phenotypes with value set definition conciseness and time to create.

Condition name			SNOMED CT concepts to define									Time to create							
			Extensional^a^ (n)		Intensional (n)		Ratio to define (download)		Extensional^b^ (n)		Ratio to define (derived)		Extensional^b^ (min)		Intensiona (min)		Diff min		Ratio (ext/int)^c^
CKD-5^d^ and ESRD^e^			5		3		1.7		27		9		17		3		12		5
Hypertension			12		3		4		233		77.7		101		3		81		27.9
Kidney transplant			8		8		1		36		4.5		19		15		3		1.2
Pregnancy, narrow			35		6		5.8		156		26		69		4		54		14.5
Pregnancy, broad^f^			35		20		1.8		1262		63.1		531		12		415		35.6
Bipolar disorder			99		2		49.5		109		54.5		49		5		37		8.5
Depression and dysthymia			72		2		36		151		75.5		67		5		51		11.3
Personality disorders			26		2		13		48		24		24		5		17		4.4
Diabetes			89		6		14.8		441		73.5		188		6		147		25.5
Prostate cancer			12		3		4		19		6.3		10		7		5		1.8
Pain related to prostate cancer			17		14		1.2		20		1.4		12		7		6		1.8
Overall sum, ratio, or %			375		49		7.5		1240		25.3		556		60		413		7.9
**Set of 10 conditions**																			
	Overall median	21.5		3		4.9		78.5		25		36.7		5		27.2		6.7	
	Overall minimum	5		2		1		19		1.4		10		3		3		1.2	
	Overall maximum	99		14		49.5		441		77.7		188		15		147		27.9	
	Overall range	94		12		48.5		422		76.2		178		12		144		26.7	

^a^Download from Value Set Authority Center.

^b^Derived from intensional.

^c^Ext/int: extensional/intensional

^d^CKD-5: chronic kidney disease, stage 5.

^e^ESRD: end-stage renal disease.

^f^Not included in summary calculated measures (overall sum, median, minimum, maximum, range).

For the full extensional value sets derived from the intensional rules, the median number of concepts-to-define was 78.5 concepts. The median ratio of concepts needed to fully define an equivalent extensional value set was 25 times that needed for the intensional value set rule.

**Figure 1:**
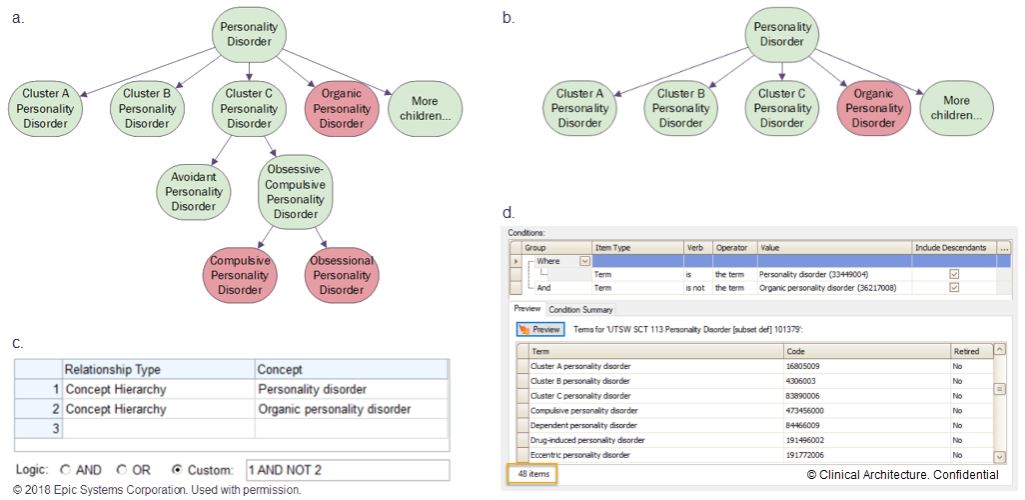
(a) SNOMED CT extensional value set list (26 items) downloaded for the condition personality disorder, shown as green-colored members of a SNOMED CT hierarchy (partial diagram only). Red-colored items aren’t on the list, downloaded from the Value Set Authority Center. (b) Matched intensional value set combining SNOMED CT hierarchies with Boolean logic: personality disorder (disorder; 33449004), including descendants AND NOT organic personality disorder (disorder; 36217008), including descendants. (c) Implementation of intensional value set in an electronic health record (EHR) (Epic Systems). (d) Implementation of intensional value set in an EHR-agnostic terminology software program (Symedical). Also shown is part of the exactly equivalent extensional value set (containing 48 SNOMED CT concepts), automatically derived from the intensional logic.

As one example, the clinical phenotype of personality disorder is specified by 26 SNOMED CT concepts in the downloaded extensional value set (Figure 1). In contrast, the corresponding intensional value set rule (inferring intent of subtypes desired from examining the VSAC downloaded list) includes just two concepts: (1) Personality disorder (SCT ID 33449004), including descendants, AND NOT (2) Organic personality disorder (SCT ID 36217008), including descendants. This rule includes all 26 SNOMED CT concepts in the VSAC extensional value set plus an additional 22 closely related concepts that reasonably belong, for 48 included concepts and a concept ratio-to-define of 48/2 or 24.0.

### Time to Create

Not surprisingly, more concise value sets are easier and faster to construct, perform quality assurance on, review, and update as needed. As shown in Table 1, it takes 6 to 8 times longer to construct an extensional value set completely equivalent in contents to an intensional value set (median 6.7, overall ratio 7.9). In this set, creating intensional value sets (groupers) for all 10 conditions was accomplished in just 1 hour (60 minutes) of keyboard time, while creating the equivalent extensional value sets required nearly 11 hours (650 minutes). The median creation time for these 10 conditions was 5 minutes for an intensional value set and 37 minutes for an equivalent extensional value set.

### Completeness: SNOMED CT Concepts

SNOMED CT is updated twice yearly [18] and an intensional rule-based approach presumably should be more resilient to updates by automatically including new descendants within an existing included hierarchy, for instance. Accordingly, we examined the relative completeness of downloaded extensional versus corresponding intensional value sets.

**Figure 2:**
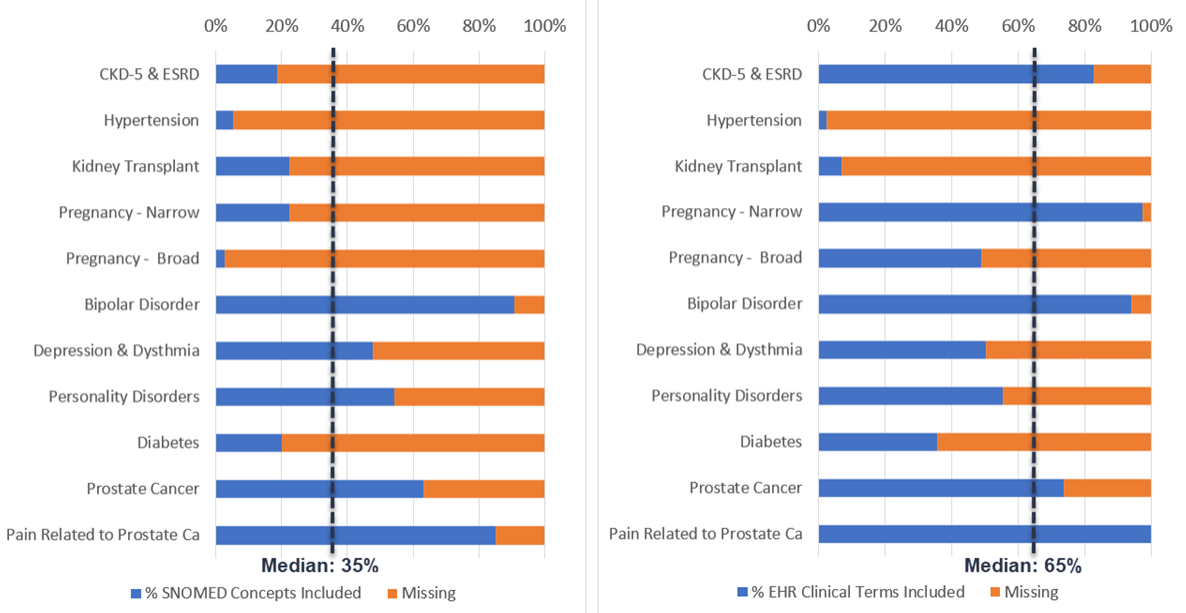
Left: SNOMED CT concepts included in 2018 extensional value sets as a percentage of those in intensional value sets. Right: Electronic health records clinical terms included using the 2018 extensional value sets as a percentage of those using intensional value sets. CKD-5: chronic kidney disease, stage 5; ESRD: end-stage renal disease; Ca: cancer.

Table 2 compares the number of SNOMED CT concepts included in the full extensional list derived from the intensional rule versus the extensional 2018 list downloaded from VSAC. Across the 10 conditions, the full derived list included a median of 3.3 times as many SNOMED CT concepts as the corresponding downloaded list (range 1.1 to 19.4). In percentage terms, a median of only 35% of SNOMED CT concepts in the full derived extensional list were present in the corresponding downloaded extensional list (range 5% to 91%), as shown in Figure 2 (left panel).

The vast majority of discrepancies between the two sets of extensional lists (877/889, 98.7%) were present in the intensional-derived list only and missing from the VSAC-downloaded list (Table 2); 1.3% (12/889) of concepts in the VSAC download were not in the intensional-derived list. Of these 12, 6 were kidney transplant procedural concepts rather than disorder or condition concepts and had no corresponding diagnosis clinical terms defined in the EHR’s clinical terminology. The remaining 6 were judged clinically relevant omissions from the intensional-derived list.

### Completeness: Coverage of Relevant Electronic Health Record Clinical Terms

Pragmatic clinical trials, registries, and other research projects that rely on EHR data for clinical phenotypes need the most accurate and complete value sets possible to define primary and comorbid conditions. We thus compared the number of EHR clinical terms (sourced from IMO, overall n>800,000) selectable by clinicians that are in extensional versus intensional value set compiled lists (see Table 3 and Figure 2, right panel).

In 9 of 10 conditions, the number of EHR clinical terms identified using the downloaded extensional value set was less than when using the corresponding intensional value set, in some cases dramatically so. In this subset of 10 conditions, a median 65% of the EHR diagnostic clinical terms selectable by clinicians in a commonly used EHR are included when using a published list-based extensional value set compared with using a corresponding concept hierarchy-based intensional value set. That is, a median of 35% of clinician-selectable diagnosis terms in the EHR for defining a clinical phenotype are missing when using a 2018 downloaded extensional value set.

**Table 2:** Comparison of downloaded versus derived SNOMED CT value set contents.

Condition name		SNOMED CT concepts included in value set				Extensional concept discrepancies		
		Extensional^a^ (n)	Extensional^b^ (n)	Ratio (derived/ VSAC)	SNOMED CT concepts included in VSAC download (%)	Total (n)	Only in VSAC download (n)	Only in intensional-derived (n)
CKD-5^c^ and ESRD^d^		5	27	5.4	19	22	0	22
Hypertension		12	233	19.4	5	223	1	222
Kidney transplant		8	36	4.5	22	40	6	34
Pregnancy, narrow		35	156	4.5	22	121	0	121
Pregnancy, broad^e^		35	1262	36.1	3	1227	0	1227
Bipolar disorder		99	109	1.1	91	10	0	10
Depression and dysthymia		72	151	2.1	48	79	0	79
Personality disorders		26	48	1.8	54	22	0	22
Diabetes		89	441	5	20	358	3	355
Prostate cancer		12	19	1.6	63	11	2	9
Pain related to prostate cancer		17	20	1.2	85	3	0	3
Overall sum, ratio, or %		375	1240	3.3	30	889	12	877
**Set of 10 conditions**								
	Overall median	21.5	78.5	3.3	35	31	0	28
	Overall minimum	5	19	1.1	5	3	0	3
	Overall maximum	99	441	19.4	91	358	6	355
	Overall range	94	422	18.3	86	355	6	352

^a^Download from Value Set Authority Center (VSAC).

^b^Derived from intensional.

^c^CKD-5: chronic kidney disease, stage 5.

^d^ESRD: end-stage renal disease.

^e^Not included in summary calculated measures (overall sum, median, minimum, maximum, range).

**Table 3:** Number of diagnosis clinical terms selectable by clinicians in the electronic health record by source of value set.

Condition name		Extensional^a^ (n)	Intensional (n)	Ratio (intensional/VSAC)	EHR^b^ clinical terms included in VSAC download (%)
CKD-5^c^ and ESRD^d^		485	586	1.2	83
Hypertension		131	5473	41.8	2
Kidney transplant		49	708	14.4	7
Pregnancy, narrow		23,429	24,043	1	97
Pregnancy, broad^e^		23,429	47,812	2	49
Bipolar disorder		1640	1744	1.1	94
Depression and dysthymia		978	1946	2	50
Personality disorders		401	724	1.8	55
Diabetes		11,997	33,707	2.8	36
Pain related to prostate cancer		149	149	1	100
Overall sum, ratio, or %		39,445	69,332	1.8	57
**Set of 10 Conditions**					
	Overall median	443	1234	1.6	65
	Overall minimum	49	149	1	2
	Overall maximum	23,429	33,707	41.8	100
	Overall range	23,380	33,558	40.8	98

^a^Download from Value Set Authority Center (VSAC).

^b^EHR: electronic health record.

^c^CKD-5: chronic kidney disease, stage 5.

^d^ESRD: end-stage renal disease.

^e^Not included in summary calculated measures (overall sum, median, minimum, maximum, range).

## Discussion

### Principal Results

For 10 conditions referenced by the CMS 2018 high-priority clinical quality measures, we compared extensional SNOMED CT lists of codes downloaded in the fall of 2018 from the VSAC with intensional (rule-based) value sets for the same conditions. Intensional value set definitions were far more concise (median number of concepts needed for equivalent value sets 3 vs 75), faster to construct (median 5 vs 37 minutes each), and more complete. VSAC-downloaded value sets were missing a median 65% of the SNOMED CT concepts included in the intensional rule-based value sets and 35% of the mapped diagnosis clinical terms selectable by clinicians within the EHR.

The conciseness of intensional value sets expedites construction in the EHR. This should also streamline vetting with busy clinical experts and harmonizing multiple value set specifications of the same real-world condition. Many systems will directly import large extensional value set files, mitigating the value set construction time/workload difference for those organizations. Still, someone must create the source value sets initially and periodically update them, and some customer organizations will have to enter them manually as well. For those, the large time reduction offered by intensional value set definitions remains an advantage. Because intensional value sets are rule-based and can include references to all descendants of a parent concept, they are more likely to include all relevant concepts than an enumerated list. That is, they are less likely to inadvertently omit descendant concepts and more likely to remain complete following future SNOMED CT updates.

### Clinical Phenotyping for Clinical-Translational Studies Using Electronic Health Record Data

Pragmatic clinical trials, registries, and other clinical and translational research studies employing EHR data for computable clinical phenotypes (rather than manual abstraction) rely on having as accurate and complete value sets to define primary and comorbid conditions as feasible [19,20]. Concern typically arises about missing diagnosis data not yet entered in the EHR by clinicians on the patient’s Problem List or as Encounter Diagnoses. While Problem List completeness in particular remains a subject of active inquiry and improvement efforts [21-26], this study raises a different concern for completeness of value set definitions when physicians and advanced practice providers have conscientiously recorded their patients’ specific diagnoses in the EHR. In this subset of 10 conditions, a median 35% of the EHR diagnostic clinical terms selectable by clinicians in a commonly used EHR are missing when using a published extensional value set compared with using a simpler rule-based intensional value set. Patients for whom those missing EHR terms are selected by clinicians will fail to be included in the selected population with the clinical phenotype. Controlling for comorbid conditions in multivariable modeling will similarly be negatively impacted by missing clinical EHR terms. Defining clinical phenotypes more completely with rule-based intensional value sets leveraging SNOMED CT’s hierarchical structure advances the feasibility and reliability of pragmatic clinical studies and learning health care system cycles conducted with EHR data produced during normal clinical care [27-29].

### Analytic Interoperability for Population Health

With the expansion of clinically integrated networks and cross-institution specialty registries to provide and measure value-based care, definition of subpopulations of patients becomes crucial for risk assessment and tailored interventions [1,30-32]. Many networks encompass a variety of EHRs. Since the designated interoperability language between EHRs for diagnoses (conditions) is SNOMED CT, employing SNOMED CT value sets enables EHR-agnostic consistent definition of subpopulations for registries, clinical decision support to promote best practices within the EHR, care gap closure, and quality measurement [9]. This provides analytic interoperability across disparate EHRs even if using clinical terminologies from different vendors. The populations that would most benefit from intervention may change over time, thus generating requests for new computable clinical phenotype definitions. The conciseness and clinical understandability of intensional value sets streamline rapid-cycle definition and vetting by specialists, as well as more facile and consistent implementation across a broad range of EHRs, population health tools, and clinical settings. These advantages make employing SNOMED CT concept rule-based intensional value sets a higher quality, better fit-for-purpose method for defining computable clinical phenotypes for population health than traditional extensional lists.

### Authoring Practice Guidelines and Electronic Clinical Quality Measures for Streamlined Implementation

With the expansion in medical knowledge and appreciation of the complexities of achieving optimal care for subpopulations of patients with a wide variety of conditions, the number of clinical practice guidelines continues to grow [33,34]. Significant effort and expense (in terms of experts’ time) goes into writing consensus guidelines and optimal practices for a condition. Achieving real-world practice change takes a long time and is often incomplete [35-37]. EHR-based clinical decision support has been shown to improve clinical process measures across multiple clinical domains [38-48].

Yet current guidelines can be difficult to implement as point-of-care clinical decision support to help “make the right thing the easy thing to do” for busy clinicians within their daily work tool, the EHR [49-53]. Non–value-added work can include:

- Translating prose definition of conditions covered by guideline, conditions excluded, and comorbid conditions into value sets implementable in EHRs to cover clinical terms/codes present in EHRs in practice
- Translating prose definitions of medication types and/or procedure types into EHR-implementable value sets
- Translating prose descriptions (and some flow charts and decision trees, if constructed ambiguously) into implementable decision algorithms for clinical decision support logic [54]

EHRs have local codes that can hamper implementation, but increasingly these are mapped to standard terminology codes to achieve interoperability with other EHRs as organizations participate in health information exchanges [27].

**Figure 3:**
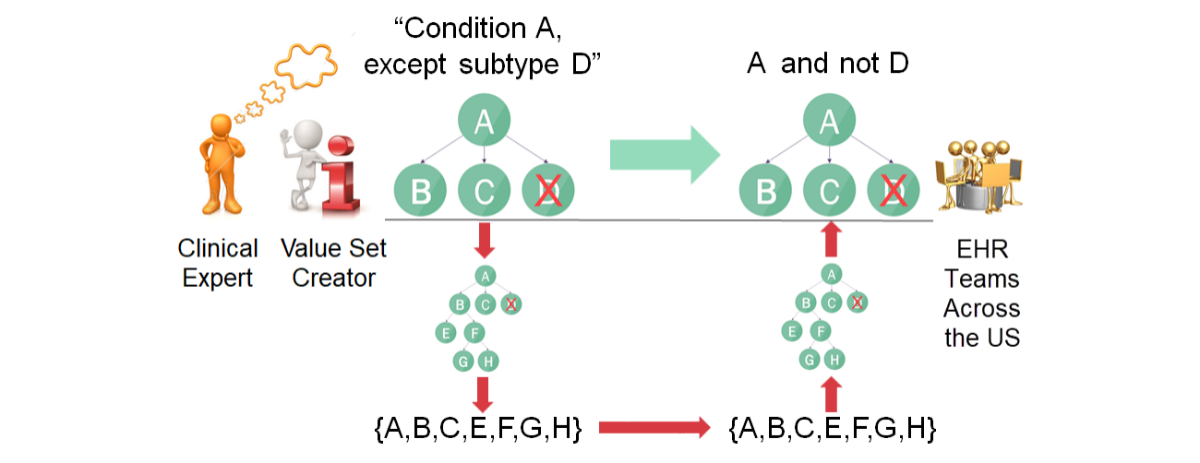
Clinicians’ thinking about type/subtype inclusion criteria matches SNOMED CT concept hierarchy implementation in electronic health records (EHRs). Deriving flat code lists (red arrows) requires reverse engineering by each EHR team to achieve the benefits of a concept hierarchy-based definition.

To accelerate implementation, we propose that specialty guideline and eCQM writing committees include a medical informaticist (as either a consultant or a formal member of the writing group representing a clinical informatics specialty society). During initial guideline development discussion and through subsequent detailed specification, the medical informaticist could then assist specialist experts on the committee in expressing the clinical conditions relevant to the guideline or eCQM in a SNOMED CT supertype-subtype form, readily implementable in an EHR or other internet-accessible repository as a concise, easily shareable intensional rule (Figure 3).

Doing so would avoid the considerable extra work of constructing a de novo extensional value set, vetting the full list with clinical experts, distributing it, and having multiple teams of EHR analysts and clinical informaticists around the country independently reverse-engineer the list into a supertype-subtype rule-based form to gain its benefits of conciseness, maintainability, completeness, and understandability for their local EHR implementation. In lean terms, that extra work (red arrows in Figure 3) could be considered non–value-adding waste. In contrast, coproducing concise, shareable SNOMED CT intensional value sets contemporaneously with the guideline and/or eCQM specification would expedite practical dissemination of clinical decision support to promote the new best practice in a consistent subpopulation of patients across the country, matching the guideline writing specialists’ intent.

### Limitations

#### Changes to SNOMED CT

Importantly, although intensional value sets retain accuracy and completeness across many updates to SNOMED CT’s contents, they are not impervious to changes [55-57]. With intensional rules referencing SNOMED CT’s hierarchical structure, additions of new descendants are generally automatically included. Some value sets may need clinical vetting for updates after new SNOMED CT releases, perhaps particularly when an intensional rule includes some, but not all, of a parent concept’s children. To enhance rapid re-vetting when needed, automated detection of new SNOMED CT concept additions that are within the span of a given rule-based grouper would be useful. One question to explore further is whether a specific inclusion strategy (include these specific siblings) versus an exclusion strategy (include all the children of the parent except these specific children) proves more resilient (remains more complete and accurate).

Migrations of existing SNOMED CT concepts to a different location in the hierarchy due to cleanup of SNOMED CT quality issues [58] pose a different challenge, although in many cases an intensional value set will handle the correction gracefully [9]. As clinicians and medical informaticists work with intensional value sets to define important clinical phenotypes, iterative improvements in SNOMED CT’s hierarchical arrangement will likely ensue, following the data quality aphorism “what gets used, gets better” [1].

#### Scope of This Paper’s Analysis

One limitation of this paper is that the comparable intensional value sets were developed and vetted only at one institution (University of Texas Southwestern Medical Center) and cannot be considered to represent national specialty society views. However, our experience demonstrates feasibility for a medical informaticist to build an initial candidate rule for defining a condition, then identify any clinical inclusion/exclusion questions for vetting with a clinician specializing in the condition [9]. For multi-institutional and/or specialty society vetting, a Modified Delphi technique can be employed as was successfully used by Buchanan [59] previously to gain working consensus across institutions. Our vision is that increasingly intensional value sets are produced as a byproduct of clinical guideline and eCQM authoring, dramatically reducing the need for individual institutions to reinvent the wheel (Figure 3).

This study only covers 10 conditions and may not be representative of all and so should be considered merely as a deep dive into one set of conditions for CMS-designated eCQMs (as described in the Methods section). We took a conservative approach in matching intensional definitions to VSAC-downloaded extensional definitions, otherwise differences reported between intensional and extensional value set completeness would have been even greater. Specifically, for pregnancy we did not use our existing broad pregnancy intensional value set: instead we constructed a new, much more narrowly defined value set intended to match the scope of the VSAC-released pregnancy value set. Similarly, for pain related to prostate cancer, we lacked an existing intensional value set and constructed our new intensional value set closely mirroring the contents of the VSAC extensional value set. Both result in minimizing differences between the extensional and intensional approaches. Given the high percentage of missing concepts and clinical terms in conditions with large numbers of terms (hypertension), our prespecified use of medians instead of means (averages) also reduced the magnitude of the reported difference between intensional and extensional approaches.

### Conclusions

Although extensional lists of codes have long been used for ICD-based value sets, the use of extensional lists of SNOMED CT codes is suboptimal and fails to leverage the capabilities and clinical relevance of ontological relationships within SNOMED CT. Compared with SNOMED CT extensional (list) value sets, intensional (rule-based) value sets are far simpler to create, maintain, understand, and vet with specialist clinicians. For the 10 conditions studied here from the 2018 CMS high-value eCQMs for the MIPS program, intensional value sets also proved substantially more complete than their corresponding extensional list versions: a median 35% of diagnosis terms selectable in the EHR by clinicians were missing when using a downloaded extensional value set, with risk of failing to identify patients with a given clinical phenotype despite physician-entered discrete diagnoses in the EHR.

Consequently, in the EHR era we believe defining conditions as computable clinical phenotypes preferentially should employ SNOMED CT concept hierarchy-based (intensional) value sets rather than extensional lists. By doing so, clinical guideline and eCQM authors can streamline broad EHR implementation of condition-specific decision support promoting guideline adherence and patient benefit.

## Appendix

Multimedia Appendix 1 Source electronic clinical quality measures and corresponding VSAC-downloaded extensional value sets in MS Excel format.

Multimedia Appendix 2 Intensional value set definitions with corresponding derived extensional value sets in MS Excel format.

Multimedia Appendix 3 Study data for tables and analyses in MS Excel format.

## Abbreviations

CKD-5: chronic kidney disease, stage 5
CMS: Centers for Medicare and Medicaid Services
ESRD: end-stage renal disease
eCQM: electronic clinical quality measure
EHR: electronic health record
ICD: International Classification of Diseases
IMO: Intelligent Medical Objects
MIPS: Merit-Based Incentive Payment System
NIH: National Institutes of Health
VSAC: Value Set Authority Center
