# Correction: SNOMED CT Concept Hierarchies for Computable Clinical Phenotypes From Electronic Health Record Data: Comparison of Intensional Versus Extensional Value Sets

**DOI:** 10.2196/14654

**Published:** 2019-07-11

**Authors:** Ling Chu, Vaishnavi Kannan, Mujeeb A Basit, Diane J Schaeflein, Adolfo R Ortuzar, Jimmie F Glorioso, Joel R Buchanan, Duwayne L Willett

**Affiliations:** 1 University of Texas Southwestern Medical Center Dallas, TX United States; 2 University of Wisconsin School of Medicine and Public Health Madison, WI United States

The authors of “SNOMED CT Concept Hierarchies for Computable Clinical Phenotypes From Electronic Health Record Data: Comparison of Intensional Versus Extensional Value Sets” (J Med Internet Res 2019;7(1):e11487) have recognized that during the final pre-publication process the copyeditors were inadvertently provided the pre-review version of the manuscript rather than the revised version of the manuscript accepted after peer review. Accordingly, a number of changes have been made to restore content of the correct version of the manuscript.

1. In the “Overview” subsection within the Introduction, a new sentence has been added to the start of the third paragraph. The first sentence of that paragraph had been:
  In this study, we examined value sets defining 10 conditions referenced by 2018 Centers for Medicare and Medicaid Services (CMS) high-priority electronic clinical quality measures (eCQMs) for adults.
  Now, the first two sentences of that paragraph are as follows:
  In the United States, the governmental Centers for Medicare and Medicaid Services (CMS) employs public quality measures to help assure the quality of health care for Medicare beneficiaries, primarily the elderly or disabled. In this study, we examined value sets defining 10 conditions referenced by 2018 Centers for Medicare and Medicaid Services (CMS) high-priority electronic clinical quality measures (eCQMs) for adults.
2. In the “Procedures” subsection within the Methods, two sentences have been added to the end of the second paragraph. In addition, the verb “create” has been replaced with “construct” in the previously last sentence, to be consistent with usage in the rest of the manuscript.
  That second paragraph had ended with the following sentence:
  Identically matching intensional value sets were then created in Symedical (in addition to Epic), and the time to create each intensional value set recorded.
  Now, the second paragraph's final three sentences are:
  Identically matching intensional value sets were then constructed in Symedical (in addition to Epic), and the time to construct each intensional value set recorded. Intensional value sets were defined using a “search, drill-up, drill-down” approach previously described [9]. Existing and newly-defined intensional value sets were vetted by medical informaticians and clinicians by deriving the full list of included SNOMED CT concepts for review.
3. The measure name “time to create” has been changed to “time to construct” in each place it was used, as follows:
  - In Methods, in the “Measures and Outcomes” subsection, the 2nd sub-subsection has been renamed from “Time to Create” to “Time to Construct”
  - In the contents of this same 2nd sub-subsection (now “Time to Construct”), two types of changes have been made:
    - A new paragraph has been inserted at the start of the subsection to explain the purpose of the “Time to Construct” measure.
    - Each mention of “time to create” has been replaced with “time to construct” in the remainder of this subsection.
  - Additionally, the location of the phrase “in Symedical” in the former first sentence of the first paragraph has been moved earlier in that sentence for improved readability, without changing the intended meaning. Thus, this subsection as a whole has been changed from the following two paragraphs:
    ***Time to Create***
    The time to create each of 11 intensional value sets (including both pregnancy value set versions) as well as 3 of the extensional value sets (CKD-5 & ESRD; prostate cancer; pain related to prostate cancer) in Symedical was measured.  From this a best-fit linear equation was derived: time (min) = 0.4177*(# SNOMED CT concepts) + 3.8707. This corresponds to an obligate time of just under 4 minutes to create any value set (eg, for configuring basic common settings), plus approximately 0.42 minutes (25 seconds) to add each SNOMED CT concept. The time to create the remaining extensional value sets was estimated using this equation.
    The difference in time to create an extensional versus an intensional value set was calculated as (time to create extensional value set) – (time to create intensional value set), expressed in minutes. The dimensionless ratio was calculated as (time to create extensional value set) / (time to create intensional value set).
    To the following three paragraphs:
    ***Time to Construct***
    The purpose of the “Time to Construct” measure is to gauge the time needed at each healthcare organization to construct in their local systems, such as their EHR, an approved value set definition received from a defining group such as VSAC (or a local clinical terminology committee). The preceding upfront “time to define” the value set, including iterative clinical review, is purposefully not included.
    The time to construct in Symedical each of 11 intensional value sets (including both pregnancy value set versions) as well as 3 of the extensional value sets (CKD-5 & ESRD; prostate cancer; pain related to prostate cancer) was measured.  From this a best-fit linear equation was derived: time (min) = 0.4177*(# SNOMED CT concepts) + 3.8707. This corresponds to an obligate time of just under 4 minutes to construct any value set (eg, for configuring basic common settings), plus approximately 0.42 minutes (25 seconds) to add each SNOMED CT concept. The time to construct the remaining extensional value sets was estimated using this equation.
    The difference in time to construct an extensional versus an intensional value set was calculated as (time to construct extensional value set) – (time to construct intensional value set), expressed in minutes. The dimensionless ratio was calculated as (time to construct extensional value set) / (time to construct intensional value set).
  - In Results, the title of the third subsection has been changed from “Time to Create” to “Time to Construct”.
  - In this same subsection (now “Time to Construct”), each instance of “time to create” has been replaced with “time to construct”.
    Accordingly the final two sentences of this subsection have been changed from:
    In this set, creating intensional value sets (groupers) for all 10 conditions was accomplished in just 1 hour (60 minutes) of keyboard time, while creating the equivalent extensional value sets required nearly 11 hours (650 minutes). The median creation time for these 10 conditions was 5 minutes for an intensional value set and 37 minutes for an equivalent extensional value set.
    To:
    In this set, constructing intensional value sets (groupers) for all 10 conditions was accomplished in just 1 hour (60 minutes) of keyboard time, while constructing the equivalent extensional value sets required nearly 11 hours (650 minutes). The median construction time for these 10 conditions was 5 minutes for an intensional value set and 37 minutes for an equivalent extensional value set.
  - In Table 1, two instances of “time to create” have been changed to “time to construct”:
    - The Table title has been changed from “Clinical phenotypes with value set definition conciseness and time to create.” to “Clinical phenotypes with value set definition conciseness and time to construct.”
    - The right-most top-level column heading has been changed from “Time to create” to “Time to construct”.
4. In the “Limitations” subsection of the Discussion, the sub-subsections below Level 2 have been reorganized.
  Previously this had two sub-subsections:
  - Changes to SNOMED CT
  - Scope of This Paper's Analysis
  Now, the structure of the “Limitations” subsection is as follows:
  - Limitations
    - Challenges When Using SNOMED CT
      - Navigating the SNOMED CT Hierarchy and Selecting Concepts for an Intensional Value Set
      - Changes to SNOMED CT
    - Scope of This Paper’s Analysis and Differences in Value Set Intent
5. Additional text has been added under the new heading “Navigating the SNOMED CT Hierarchy and Selecting Concepts for an Intensional Value Set” as follows:
  Because of the polyhierarchical structure of SNOMED CT, potential exists for inadvertently including descendant branches and/or individual concepts which do not belong. The “search, drill-up, drill-down” approach employed mitigates that risk by explicitly exploring if the currently-selected concept in a SNOMED CT hierarchy browser is too general or too narrow [9]. A helpful additional mitigation strategy is to expand the intensional rule to show all included SNOMED CT concepts as a derived extensional list (we used Symedical for this purpose), then having a clinician view this list for any additional concepts which should be excluded. These then similarly can be evaluated with the “search, drill-up, drill-down” method to find the optimal concept in the hierarchy for exclusion along with its descendants.
6. The text content under the Level 3 heading previously named “Scope of This Paper's Analysis” and now renamed “Scope of This Paper’s Analysis and Differences in Value Set Intent” has been updated as follows:
  - The first paragraph is unchanged.
  - In the second paragraph, the final sentence has been deleted. The paragraph previously ended with the following two sentences:
    Both result in minimizing differences between the extensional and intensional approaches. Given the high percentage of missing concepts and clinical terms in conditions with large numbers of terms (hypertension), our prespecified use of medians instead of means (averages) also reduced the magnitude of the reported difference between intensional and extensional approaches.
    Now the paragraph ends with:
    Both result in minimizing differences between the extensional and intensional approaches.
  - Two new paragraphs have been added to the end of this section:
    On the other hand, for hypertension our existing intensional value set includes all forms of hypertension (meant to represent the scope covered by recent hypertension guidelines [60-62]), whereas the VSAC-downloaded extensional value set was specific to essential (primary) hypertension. The latter did not include SNOMED CT concepts for the general concept of “Hypertensive disorder, systemic arterial (disorder)” not specified to be primary or secondary, for secondary hypertension, or for “Complication of systemic hypertensive disorder (disorder)”. Replacing our existing hypertension intensional value set with one mirroring the contents of the VSAC-downloaded essential hypertension value set would have increased this condition’s values for % completeness of both SNOMED CT concepts and EHR terms.  However, our pre-specified use of medians instead of means (averages) results in no change in the overall median values reported of 35% completeness for SNOMED CT concepts and 65% completeness for EHR clinical terms.
    Inconsistencies in SNOMED CT polyhierarchy “is a” definitions may lead to inadvertent inclusion of unwanted descendants of a seemingly wholly-appropriate SNOMED CT concept. Use of the “search, drill-up, drill-down” method during intensional value set definition can reduce the likelihood of this, as can clinical review of the full list of included SNOMED CT concepts derived from the intensional definition [9]. As discovered, such unwanted descendants can be specifically excluded in the intensional rule. Also requests to update the “is a” relationship in SNOMED CT to a more specific parent(s) can be made through the SNOMED CT Content Request Service.  Once the subsumption has been updated in SNOMED CT, the value set intensional rule typically can be further simplified.
  Three new references have been added [60-62], referred to within the two new paragraphs added above. The added references are:
  60. James PA, Oparil S, Carter BL, Cushman WC, Dennison-Himmelfarb C, Handler J, et al. 2014 evidence-based guideline for the management of high blood pressure in adults: report from the panel members appointed to the Eighth Joint National Committee (JNC 8). JAMA 2014 Feb 05;311(5):507-520. [doi: 10.1001/jama.2013.284427] [Medline: 24352797]
  61. Whelton PK, Carey RM, Aronow WS, Casey DE, Collins KJ, Dennison Himmelfarb C, et al. 2017 ACC/AHA/AAPA/ABC/ACPM/AGS/APhA/ASH/ASPC/ NMA/PCNA Guideline for the Prevention, Detection, Evaluation, and Management of High Blood Pressure in Adults: A Report of the American College of Cardiology/American Heart Association Task Force on Clinical Practice Guidelines. J Am Coll Cardiol 2018 May 15;71(19):e127-e248 [FREE Full text] [doi: 10.1016/j.jacc.2017.11.006] [Medline: 29146535]
  62. Williams B, Mancia G, Spiering W, Agabiti Rosei E, Azizi M, Burnier M, ESC Scientific Document Group. 2018 ESC/ESH Guidelines for the management of arterial hypertension. Eur Heart J 2018 Sep 01;39(33):3021-3104. [doi: 10.1093/eurheartj/ehy339] [Medline: 30165516]

The correction will appear in the online version of the paper on the JMIR website on July 11, 2019, together with the publication of this correction notice. Because this was made after submission to PubMed, PubMed Central, and other full-text repositories, the corrected article also has been resubmitted to those repositories.

